# Multifunctional wide-angle optics and lasing based on supercell metasurfaces

**DOI:** 10.1038/s41467-021-24071-2

**Published:** 2021-06-18

**Authors:** Christina Spägele, Michele Tamagnone, Dmitry Kazakov, Marcus Ossiander, Marco Piccardo, Federico Capasso

**Affiliations:** 1 Harvard John A. Paulson School of Engineering and Applied Sciences, Harvard University, Cambridge, MA USA; 2 Fondazione Istituto Italiano di Tecnologia, Genova, Italy; 3 CNST – Fondazione Istituto Italiano di Tecnologia, Milan, Italy

**Keywords:** Lasers, LEDs and light sources, Metamaterials, Nanophotonics and plasmonics

## Abstract

Metasurfaces are arrays of subwavelength spaced nanostructures that can manipulate the amplitude, phase, and polarization of light to achieve a variety of optical functions beyond the capabilities of 3D bulk optics. However, they suffer from limited performance and efficiency when multiple functions with large deflection angles are required because the non-local interactions due to optical coupling between nanostructures are not fully considered. Here we introduce a method based on supercell metasurfaces to demonstrate multiple independent optical functions at arbitrary large deflection angles with high efficiency. In one implementation the incident laser is simultaneously diffracted into Gaussian, helical and Bessel beams over a large angular range. We then demonstrate a compact wavelength-tunable external cavity laser with arbitrary beam control capabilities – including beam shaping operations and the generation of freeform holograms. Our approach paves the way to novel methods to engineer the emission of optical sources.

## Abstract (web summary)

The angular dependence is a well-known issue in metasurface engineering. Here the authors introduce a supercell metasurface able to implement multiple independent functions under large deflection angles with high efficiency, leading to a wavelength tunable laser with arbitrary wavefront control.

## Introduction

**Fig. 1:**
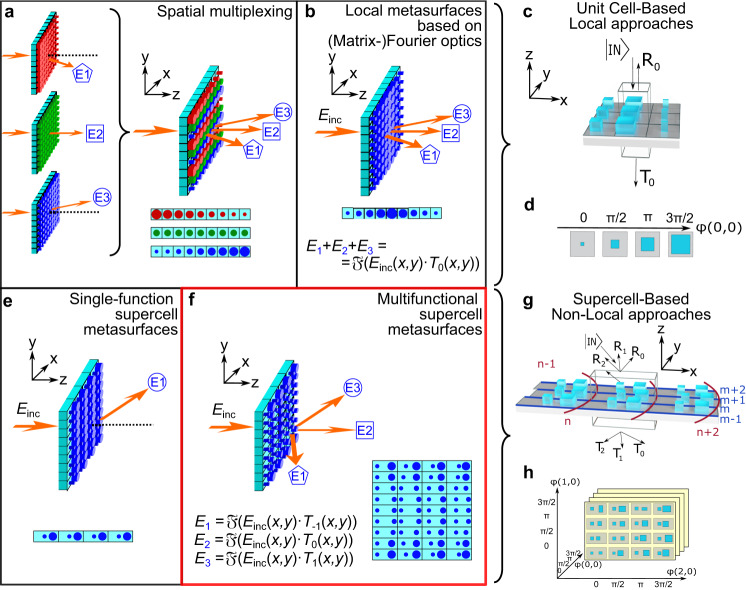
The generalized supercell metasurface concept. **a**–**c** Local metasurfaces methods. Multiple functions can be created by either **a** interleaving cells from different metasurfaces or **b** exploiting Fourier optics and adding together the amplitude and phase profiles required for each function. The complex amplitudes can be replaced by Jones matrices to achieve polarization control (Matrix Fourier Optics). Both methods have low efficiencies for large deflection angles due to the locality of their unit cells. **c** Illustration of a sub-wavelength unit cell of a local metasurface. **d** Example of traditional metasurface libraries of subwavelength unit cells that relate the locally implemented phase shifts to the physical dimensions of the metaatom. **e** Traditional supercell metasurface based on an array of supercells designed for single functions such as metagratings. **f** The proposed extended supercell metasurface (SCMS) concept based on the independent control of phase and polarization at each position and to implement independent functions on each order. The far-fields associated to each of the functions are related via the Fourier transform to the phase profile implemented on each order. **g** Unlike subwavelength unit cells, supercells possess multiple diffraction orders that depend on the coupling between the supercell elements. This non-locality becomes important as the deflection angle increases and can be rigorously taken into account in the design. **h** An example of multidimensional library of non-local supercells. The library contains a supercell for each possible combination of phases for all the orders.

The efficient transformation and shaping of light are of paramount importance for science and technology. Nowadays, modern versatile optical devices demand several functions which results in complex systems when implemented with bulk optics. In contrast, one can achieve compact, lightweight, and customized optical devices when replacing the latter with metasurfaces. Metasurfaces are artificial planar metamaterials that consist of arrays of subwavelength-spaced nanostructures, often referred to as metaatoms, which can locally manipulate amplitude, phase, and polarization of light to achieve a variety of optical functions such as lensing, structured light, enhanced cameras, and optical computing to name just a few^1–9^. However, the efficiency and the angular range of current approaches to create multifunctional metasurfaces (summarized in Fig. 1a–c) are limited, thus preventing widespread applications. Hence, there is currently no optical component with the flexibility of metasurfaces in beam shaping, which also implements different functions with large difference in deflection angle. We focus here on metasurfaces performing different functions in different directions simultaneously, as opposed to a single function that changes with different wavelengths^10,11^ or the angle of incidence^12,13^.

The two simplest approaches to multifunctional metasurfaces (Fig. 1a, b) are based on the use of local subwavelength unit cells (Fig. 1c), that are designed to impart a local phase or polarization profile to light propagating through the cell. In the simplest case, a library of unit cells can be prepared changing some physical parameters in the cell and simulating its transmission phase (Fig. 1d). By “local”, we refer to the fact that cells are designed separately and intercell coupling effects are neglected or limited to the assumption of a perfectly periodic array using Bloch or periodic boundary conditions. This causes a significant loss of efficiency and other undesired effects when large deflection angles are required. Specifically, the latter need large phase gradients which may be under-sampled by common unit cells.

Multiple functions such as focusing, holograms, polarization functions, and beam shaping can be achieved by designing separated metasurfaces (one for each function) and interleaving the cells in a compound metasurface (Fig. 1a)^14^. This approach is highly inefficient as the overall efficiency of the metasurface is roughly limited to 1/*N*, where *N* is the number of functions (see Supplementary Information for more details). Furthermore, the lost optical power forms spurious grating orders or evanescent waves along the interleaving direction. A better approach consists in recognizing that the far-field is related to the complex transmission profile of the metasurface by a Fourier transform, and exploiting the linearity of the Fourier transform by adding together the complex transmission profiles for each function (Fig. 1b). This approach can easily be extended to polarization optics^1,15^. The drawback is that even simple functions, such as creating a beam splitter that splits light into a finite number of beams with the same polarization, require both amplitude and phase to change across the metasurface and the required transmitted amplitude must locally exceed 1, implying gain, for an overall efficiency of 100% (2). This cannot be implemented with local metasurface elements.

One can overcome locality using supercells instead of unit cells^10,16–30^. Within this approach, adjacent cells are merged into a supercell containing multiple meta-atoms. These can be directly designed and simulated by considering non-local effects due to the optical coupling between the metaatoms.

When the supercell is repeated periodically, the device behaves as a grating (referred to as metagrating), splitting the light in multiple diffraction orders. Often, the function of a metagrating is to send all light in one selected diffraction order corresponding to a deflection angle, which depends on the periodicity (size) of the supercell (Fig. 1e). When the latter is varied along a radial direction, as in the case of a metalens, a phase ramp is created so that light from the corresponding order is focused to a diffraction limited spot. Because the phase is defined modulo 2π, this is equivalent to creating a saw tooth phase profile^31,32^. This approach is beneficial at the edges, where the deflection angle is larger and can be handled effectively only with supercells^16,23^. More advanced metagrating applications include polarization functions and ptychography as well as the manipulation of spin and orbital angular momentum of light^1,14,25,33–36^. However, these designs implement supercells as groups of non-interacting unit cells and hence cannot achieve large deflection angles with high transmission efficiency.

In this work, we present supercell metasurfaces (SCMS) based on a library of supercells to enable the realization of multiple functions with large deflection angles without a loss of efficiency (Fig. 1f). Using supercells as the building block of our library, we exploit the fact that the coupling between neighboring metaatoms in the supercell can re-distribute optical power within the supercell. This enables a transmission/reflection amplitude that can locally exceed unity, breaking the locality constraint of multifunctional local metasurface approaches and enabling multifunctionality with high efficiencies at large deflection angles. Each supercell in the library is optimized to achieve specific transmission phase and amplitude in each order. Different phase profiles can be implemented on each order by selecting at each position of the metasurface the supercell, that satisfies simultaneously the phase requirement for all orders. The required phase profile of a certain order can be calculated by the inverse Fourier transform of the far field of this respective function only (Fig. 1f). This allows us to demonstrate independent functions at different and large angles, such as the splitting of the incident beam into a Gaussian, a Bessel and a focused helical beam with orbital angular momentum (OAM) equal to 1, on separate diffraction orders. Importantly, our generalized approach is not bound to supercells arranged on a regular orthogonal lattice, but adds the additional degree of freedom of curvilinear lattices. The generalization includes non-local effects^37^ and exploits them in order to create the desired functionalities.

## Results

### Proposed supercell metasurface method

Our SCMS method, which generalizes previous approaches, is based on supercells arranged over a discrete lattice where each supercell is selected from a library (i.e., a supercell-library) as for metasurfaces. In local metasurfaces (Fig. 1c) the metaatom is subwavelength so the transmitted or reflected light is not diffracted in multiple orders, and the geometry of the metaatom determines the local effect of the metasurface on light. For instance, a desired phase profile is implemented by discretizing it on a lattice, and selecting the metaatom with the required phase for each position. In contrast, in a SCMS (Fig. 1g), supercells are larger and can be treated locally as a grating^30^. The size of the supercells determines the number and direction of diffraction orders to be considered, while the supercell inlay can be varied or optimized to control the complex amplitude (intensity and phase) and polarization of the respective orders independently of each other.

Therefore, the key idea of the proposed extension of supercell-metasurfaces is that independent amplitude and phase profiles can be implemented on each order by choosing at every position on the substrate a supercell from the supercell-library, that simultaneously satisfies the desired phase and amplitude requirements for all local orders. Engineering the phase profiles independently allows then different *functions* on each order, which is equivalent to implementing different effective metasurfaces on each of the orders. For a first demonstration we consider solely the transmission orders of a supercell-metasurface—the same argument will be used later for the case of reflection in the proposed external cavity application.

A traditional metasurface library relates the physical dimension of a metaatom with its local phase or polarization function (Fig. 1d). For our approach, instead, a library containing a list of supercell geometries and their respective phase and amplitude on each order can be compiled, and the supercells can be simulated with dedicated codes such as Reticolo or S4^38,39^. To illustrate this key aspect, Fig. 1h shows an example of a library which can control independently the transmission phase in multiple diffraction orders according to the supercell inlay. By trying different supercell designs, we empirically verified that supercells containing two pillars were sufficient to implement the required phases for the devices presented in this work; however, the design of the supercells can be readily extended to multiple pillars or free-form metaatoms^12,26,37,38,40–42^.

The SCMS is defined by an array of supercells, where each supercell is placed at a different point of a discrete lattice. In the simplest case, the lattice is a Bravais lattice as in a 2D crystal, which is spanned by the two lattice vectors **a** and **b**. In this case, the points are aligned along straight lines in the plane. However, in the most general case, the lattice can be chosen to be curvilinear, which has some important advantages as explained later when discussing Eq. (1). By curvilinear lattices, we mean lattices that locally can be approximated by a Bravais lattice, but at a larger scale the points are arranged on lines which can be curved. Hence each supercell can still be described and simulated as a periodic parallelogram cell, since the curvature of the curvilinear lattice becomes relevant only for distances in the order of hundreds of supercells. To describe this general case, we use two dimensionless real valued functions $a\left(x,y\right)$ and $b\left(x,y\right)$ chosen in such a way that the condition $\left\{a\left(x,y\right)=n,\quad n\;\in\;{\mathbb{Z}}\right\}$ identifies a first family of curves on the plane and similarly the condition $\left\{b\left(x,y\right)=m,\quad m\;\in\; {\mathbb{Z}}\right\}$ identifies a second family of curves. We then require each supercell to be placed at the intersection of the curves from each set, so that lattice points are points in the plane labeled by $(n,m)$. Figure 1g shows an example where the two families of curves are superimposed as red and blue on the metasurface (see Supplementary Information for details).

Because the lattice can be locally approximated as a Bravais lattice, it will locally create diffraction orders for transmitted (or reflected) light. We can label these orders with integers ${N}_{{\rm{a}}}$ and ${N}_{{\rm{b}}}$. The supercell at position (*n, m*) can therefore be described by the coefficients ${C}_{{{\rm{N}}}_{{\rm{a}}},{{\rm{N}}}_{{\rm{b}}}}\left(n,m\right)$, which indicate the complex amplitude (modulus and phase) of each of the orders when light is incident at a given angle (fixed at the beginning of the design). The supercells considered in this work do not have polarization functions, but the approach proposed here can be generalized to that case by having the coefficients ${C}_{{{\rm{N}}}_{{\rm{a}}},{{\rm{N}}}_{{\rm{b}}}}\left(n,m\right)$ that take the form of 2 × 2 Jones matrices. In the remainder of this work, we will assume the coefficients to be simple complex numbers, and that light has a fixed polarization.

Changing the local lattice spacing one can control the size of the supercells and hence the number and direction of their local orders. Let us now locally consider a metasurface as a simple linear grating with periodicity *S* along the *x* direction. The grating is effectively incrementing the in-plane component of the *k* vector of the incident light as it passes through the metasurface, so that the diffracted light acquires a phase factor ${G}_{{\rm{N}}}\left(x\right)={e}^{i\frac{2\pi N}{S}x}$ for deflection into the *N*th order. This simple observation can be immediately generalized to the case of a curvilinear grating, as

1 $${G}_{{{\rm{N}}}_{{\rm{a}}},{{\rm{N}}}_{{\rm{b}}}}\left(x,y\right)={e}^{2\pi i\left[{{\rm{N}}}_{{\rm{a}}}a\left(x,y\right)+{{\rm{N}}}_{{\rm{b}}}b\left(x,y\right)\right]}.$$

The complex transmission of the supercell metasurface ${T}_{{{\rm{N}}}_{{\rm{a}}},{{\rm{N}}}_{{\rm{b}}}}\left(x,y\right)$ can then be written as

2 $${T}_{{{\rm{N}}}_{{\rm{a}}},{{\rm{N}}}_{{\rm{b}}}}\left(x,y\right)={C}_{{{\rm{N}}}_{{\rm{a}}},{{\rm{N}}}_{{\rm{b}}}}\left(n,m\right){G}_{{{\rm{N}}}_{{\rm{a}}},{{\rm{N}}}_{{\rm{b}}}}\left(x,y\right),$$

where the coefficients ${C}_{{{\rm{N}}}_{{\rm{a}}},{{\rm{N}}}_{{\rm{b}}}}(n,m)$ describe the diffracting properties of the specific supercell at position (*n*, *m*).

The same argument applies to metasurfaces in reflection, and traditional local metasurfaces can be seen as a particular case of a supercell-metasurface where each cell is sub-wavelength and only the zeroth order (*N*_a_ = 0, *N*_b_ = 0) appears, so that Eq. 2 reduces to ${T}_{\mathrm{0,0}}\left(x,y\right)={T}_{\mathrm{0,0}}\left(n,m\right)$.

It is now clear that the advantage offered by a curvilinear lattice is to introduce an additional degree of freedom in the design. For instance, in the case of the laser described later, the focusing functionality can be implemented using only the grating effect *G*, keeping the coefficients *C* fixed. In general, the functions can be implemented using only *G* (with some limits), using only *C* on a linear lattice, or using a combination of both (and we will show examples for each case), providing a further increase in design flexibility. Of course, large-angle deflection is better implemented with grating effect *G* term, to exploit non-local effects. In this way, the *C* term becomes slowly varying across the metasurface.

Because all the supercells are simulated using periodic boundary conditions, our approach takes into account the coupling between adjacent supercells as long as these are not too different from each other. This is the case in the designs we present later (since the *C* term is slowly varying). For the same reason, during the creation of the library, we ensured that library elements which are adjacent in the performance space (here the phases space), have also similar geometrical sizes, avoiding abrupt changes in favor of smooth variations (*continuous* design). Furthermore, even in case of significant differences in adjacent supercells, the accuracy of the design can still be increased arbitrarily with larger supercells and with hierarchies of supercells (e.g., supercells of supercells, which is outside of the scope of this work).

### Multibeam super cell metasurfaces

**Fig. 2:**
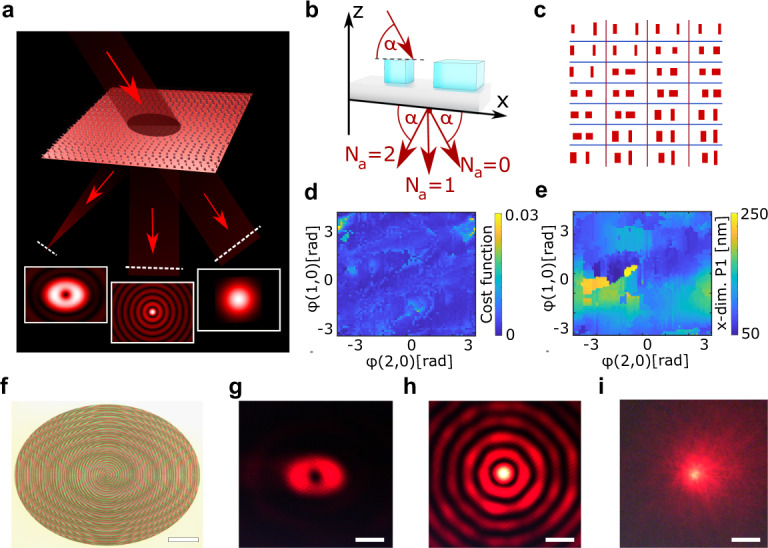
Multifunctional beam shaping supercell metasurface (SCMS). **a** Schematic and simulation results. The collimated input beam has a gaussian profile and impinges under a steep angle (52°). The SCMS splits the beam into a gaussian, a Bessel- and a focused helical beam while introducing a deflection of 0°, 52° and 104°, respectively. **b** Schematic of the two-pillar supercell. **c** Schematic of a metasurface detail. All supercells have the dimension of 800 nm × 400 nm. **d** Optimization results of the supercell library showing the cost function for a 2*π* phase coverage on the orders (1,0) and (2,0). **e** Optimization results of the supercell library showing the *x*-dimension of the first pillar for a 2*π* phase coverage on the orders (1,0) and (2,0). **f** Optical image of the fabricated SCMS. Scale bar 165 µm. **g** Optical measurement of the focused helical beam. Scale bar: 8 µm. **h** Optical measurement of the Bessel beam. Scale bar: 8 µm. **i** Optical measurement of the 0th order in the far field.

As a first example, we present a SCMS that splits a collimated, s-polarized light beam, that is incident under a large angle of 52°, into three independent beams with large difference in angle (Fig. 2a). Specifically, the zeroth order remains a gaussian beam, the first order is bend by 52° and shaped into a Bessel beam and the second is focused under an angle of 104° while imparting orbital angular momentum (OAM), creating a singularity in the focus. The simulated far fields of the orders are shown in the framed insets in the same schematic.

While the large deflection of the beams is controlled by the supercell periodicity, the functions are implemented by changing the supercell inlay at each lattice position. By choosing all supercells to have the dimension of 800 nm × 400 nm, the supercells are subwavelength in *y*, but have two additional orders in *x* (Fig. 2b). By placing the supercells on a regular grid, the metasurface will then deflect the two additional orders by the desired angles of 52° and 104°, respectively. The functions are implemented by an additional superposed phase profile, which is slowly varying and can be sampled from a library with the same phase in order (0,0) for all library elements and a 2*π* phase coverage in both additional orders (1,0) and (2,0). This library was created using a gradient descent algorithm that optimizes positions and dimensions of two pillars within the supercell, using the continuous design principle that we described earlier (Fig. 2b) (see Supplementary Information for details). Figure 2d, e are graphical representations of the cost function *f* and an example of a supercell parameter of each supercell library element with *f* defined as:

3 $$f=\mathop{\sum}\limits_{j=0}^{2}{\left|{A}_{j}\cdot {e}^{i{\phi }_{{\rm{j}}}}-{A}_{{\rm{j}},{\rm{target}}}\cdot {e}^{i{\phi }_{{\rm{j}},{\rm{target}}}}\right|}^{2}$$

with ${A}_{{\rm{j}}}$ and ${\phi }_{{\rm{j}}}$ being the simulated amplitude and phase of order *j*, and ${A}_{{\rm{j}},{\rm{target}}}$ and ${\phi }_{{\rm{j}},{\rm{target}}}$ are the corresponding desired amplitude and phase of order *j*. The optical image (Fig. 2f) shows the superposition of the characteristic spiral and the concentric rings required for a focused OAM and a Bessel beam, respectively. The measured beams are in agreement with the designed profiles (Fig. 2g–i, see Supplementary Information for measurement details).

While this example requires only two of the three supercell orders to have full phase coverage, the theory can be extended to light structuring in more orders. There is no obvious limit to the number of orders with full phase coverage, but a larger number requires the routing of the power in increasingly complex ways within the supercell. This can be achieved with designs using larger libraries with more complex supercell inlays (e.g., more than two pillars, inverse-designed-layer or multi-layer structures), which can be realized according to our theory but is beyond the scope of this paper. As a general design principle, one must ensure that the number of degrees of freedom of the supercell-design (such as size and position of pillars, etc.) is equal or larger than the number of parameters to be controlled (such as order dependent phase, amplitude, or polarization). Specifically (for the single polarization case), the number of real-valued degrees of freedom of the supercell must be at least twice the number of orders to be designed, because each order is represented by a complex number i.e., two real numbers.

### Metasurface based external cavity laser

Finally, we use this method to demonstrate experimentally a wavelength-tunable metasurface based external cavity laser (MECL). The supercell reflector design overcomes shortcomings of commonly used ECL architectures. While metasurfaces have already been used as intracavity devices to obtain orbital angular momentum lasing or to redirect the emission from solid state lasers^30,43^, or as their gain medium^44,45^, their use for external laser cavities has not been considered yet. The proposed metasurface reflector, which is tilted with respect to the diode laser source, splits the s-polarized light coming from the laser in two beams and implements on them two independent optical functions: one beam is focused back on the facet of the laser diode, providing cavity feedback and enabling laser operation, while the other is the output beam, which can be collimated or shaped arbitrarily, a functionality that otherwise would be impossible to achieve with standard metasurfaces or external cavity designs. The lasing wavelength can be controlled by moving the supercell-metasurface reflector with respect to the laser diode, without changing the direction of the output beam.

**Fig. 3:**
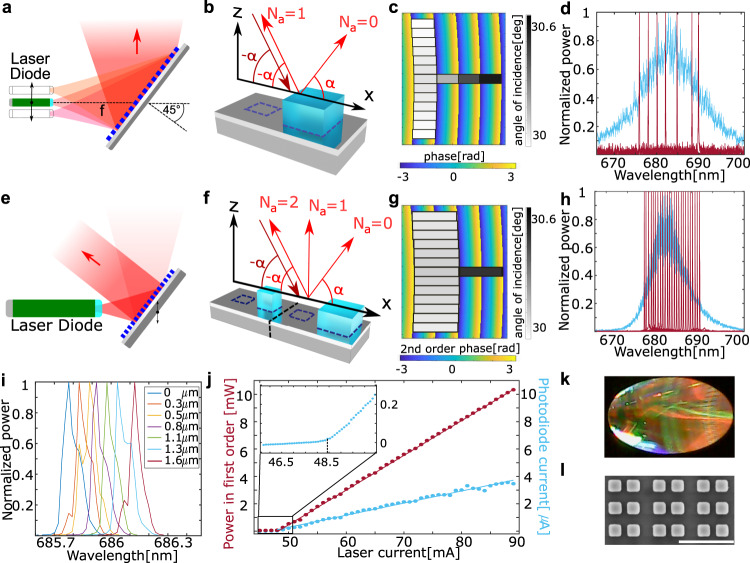
Metasurface based external cavity laser (MECL) with diverging (a–d) and collimated output (e–l). **a** Schematic of the basic MECL: a reflective metalens focuses a narrow wavelength range back on the diode facet. Changing the position of the metasurface with respect to the laser diode controls the output wavelength. **b** Schematic of a supercell containing one optical element. The angle of the first local diffraction order of each supercell matches the angle of incidence. **c** Schematic showing how the supercells (represented by rectangles) are positioned in order to create the desired phase profile in the first order. **d** Wavelength tuning for a drive current *I* = 75 mA (red) and electroluminescence spectrum for *I* = 40 mA (blue). Central wavelength = 685 nm. The achieved range corresponds to a metasurface vertical shift of 45 µm. **e** Schematic of the MECL with collimated output. **f** The new supercell is formed by two adjacent supercells shown in Fig. 2b. The intensity and phase in the first order can be controlled by creating asymmetries in these two cells (e.g., pillar size or displacement in opposite directions). **g** Schematic showing how the supercells sample the desired phase profile in the second order. **h** Normalized spectra for coarse wavelength tuning (red) and electroluminescence spectrum (blue), both for *I* = 65 mA. Central wavelength = 685 nm. **i** Fine wavelength tuning for *I* = 65 mA. Legend shows the vertical shift of the metasurface. **j** Characterization of the lasing threshold at central wavelength. **k** Optical picture of the metasurface. The metasurface major and minor axes are 5.8 mm and 2.5 mm, respectively. **l** SEM image of a metasurface detail, scale bar is 1 µm.

Metasurfaces bend light according to the designed phase profile, and they are characterized by chromatic aberrations^46^, unless they are specifically designed to avoid it. Here, we use the chromaticity to our advantage to replace the grating and the lens in a traditional external cavity laser (ECL) with an off-axis reflective metalens (tilted by 45° with respect to the laser diode axis). The latter is designed to focus the light emitted by the laser facet back to the facet itself (Fig. 3a). The reflection metasurface is based on TiO_2_ pillars on a silver substrate^11^. Due to the chromatic aberration, only a narrow wavelength range is reflected back to the AR-coated laser facet. In fact, due to the tilt of 45°, the focus of the metasurface moves as a function of the wavelength on a line perpendicular to the light propagation direction^47,48^. The laser is then tuned by varying the relative vertical position of the metasurface and the laser facet, which changes the wavelength for which the required optical feedback occurs. Small movements along the light propagation direction do not affect this wavelength selection mechanism, but allow for small adjustments of the cavity length, leading to fine tuning. Note that in contrast to conventional ECLs, where a grating rotation and translation are needed, here a 2D translation is enough for tuning.

Focusing a normally incident plane wave can be achieved if the transmission and/or reflection phase of the supercells is selected to match the following design phase profile:

4 $$\varphi \left(r\right)={k}_{0}\left(\sqrt{{r}^{2}+{f}^{2}}-f\right)+{\varphi }_{0}={k}_{0}\left(\sqrt{{x}^{2}+{y}^{2}+{f}^{2}}-f\right)+{\varphi }_{0},$$

where $\varphi \left(r\right)$ is the phase profile as function of the distance $r=\sqrt{{x}^{2}+{y}^{2}}$ from the center of the metalens, ${k}_{0}=2\pi {\lambda }^{-1}$ is the wavevector of light in free space, *f* is the metalens focal length and ${\varphi }_{0}$ is a constant arbitrary phase factor, which will not affect the performance of the metalens. In the MECL, light must be focused from one point back to itself, which can be achieved by metasurfaces operating in reflection, doubling the wavevector deflection and hence the phase profile:

5 $$\Phi =2\varphi \left(r\right)=2{k}_{0}\left(\sqrt{{r}^{2}+{f}^{2}}-f\right)+{\Phi }_{0}.$$

We implement the phase profile shown in Eq. 5 in the off-axis region defined as:

6 $$r\in \left[\sqrt{2}f{{\tan }}\left(\frac{\pi }{4}-{\alpha }_{{\rm{x}}}\right),\sqrt{2}f{{\tan }}\left(\frac{\pi }{4}+{\alpha }_{{\rm{x}}}\right)\right],$$

where ${\alpha }_{{\rm{x}}}$ corresponds to the divergence angle of the laser beam with respect to the *x*-axis and *f* to the distance between metasurface center and the laser facet.

The desired phase profile is characterized by a large gradient corresponding to a variation of the in-plane wavevector component up to 1.73 *k*_0_ upon reflection, which is caused by the large angle of both the incident and the deflected light. This makes the conventional unit-cell approach completely inadequate for the phase implementation. Most importantly, the requirement of a doubled phase gradient (Eq. 5) is very challenging and cannot be implemented with a traditional metasurface design, which would severely undersample the phase profile. In the remainder of the paper, we show that it is possible to engineer a SCMS to achieve this goal, and that it is also possible to modify the design so that only a part of the light is focused back to the laser, and the rest is collimated in an output beam which maintains its propagation direction upon wavelength tuning, and that arbitrary beam shaping is also possible.

This system has important advantages over common external cavity laser designs based on gratings, such as the Littrow and Littman-Metcalf configurations^49^. In the Littrow configuration the first grating order is used as the laser feedback, while the zero order functions as the laser output. The change in wavelength is achieved by rotating the grating and hence causes a change in direction of the output beam, which makes this configuration problematic for many applications.

The Littman–Metcalf configuration mitigates this problem by introducing a mirror into the setup. Light is deflected by a grating towards the mirror, which reflects it back to the grating. For one specific wavelength, light is orthogonal to the mirror, and hence it is reflected back on the same path and focused on the laser facet, providing feedback at that wavelength only. The lasing wavelength can be selected by rotating the mirror instead of the grating, which leaves the direction of the outgoing light unchanged. However, part of the light reflected by the mirror is lost as a specular reflection from the grating, thereby reducing efficiency to about 50% of the Littrow configuration.

The proposed system not only combines the strengths of both, Littrow and Littman–Metcalf configuration by enabling a change in wavelength, while neither introducing a change in propagation direction nor a power loss but has several additional appealing qualities. With no need for additional optical components, the proposed system is compact and easy to align. In addition, the change in wavelength of the MECL is controlled by simple translation of the metasurface, while an ECL is depending on both rotation and translation. This further eases the control of the laser. Finally, the supercell-metasurface approach enables an arbitrary control of the laser beam shape. We demonstrate the latter by presenting MECLs with diverging, collimated, or holographic outputs.

### Control of laser beams

As proof of concept, we designed an MECL that sends all light back onto the laser facet, maximizing optical feedback and using the residual metasurfaces diverging specular reflection to monitor the behavior of the laser (Fig. 3a).

Assigning the feedback beam to the first order (${N}_{{\rm{a}}}\,=\,1$) in the *x*-direction of each supercell and the output beam to their zeroth order (${N}_{{\rm{a}}}=0$), we require ${N}_{{\rm{b}}}=0$ and ${N}_{{\rm{a}}}\in \left[{\mathrm{0,1}}\right]$. The deflection angle of the first order must satisfy the retroreflection condition *α*_out_ = *α*_in_ for each supercell (Fig. 3b). The required phase profile described by Eq. 5 can be already implemented using the degree of freedom given by the curvilinear lattice. A suitable coordinate system can be chosen as:

7 $$a\left(x,y\right)=\frac{2}{\lambda }\left(\sqrt{{r}^{2}+{f}^{2}}-f\right)\quad b\left(x,y\right)=\frac{y}{U},$$

where *λ* represents the center wavelength of the active medium’s electroluminescence, *U is* the subwavelength supercell dimension in *y*-direction (chosen here to be 300 nm) to avoid diffraction orders in the orthogonal direction, *f* the focal distance of the metalens and *r* the off-axis-radius defined by Eq. 6. In this way, the focusing is controlled by the supercell length *S* that determines the direction of each local grating order. As the phase profile is controlled using the degree of freedom of the curvilinear lattice, all the supercells must have the same phase of in order to constructively interfere.

The curves ${a}_{{\rm{n}}}$ and ${b}_{{\rm{n}}}$ can be sampled by a supercell library that is composed of rectangles with dimension *S* ∈ [400 nm, 700 nm], *U* = 300 nm. One can see in Fig. 3c how the curvilinear lattice is sufficient to implement the required phase profile. As the first order deflection corresponds to a 2*π* phase ramp over the size of the supercell, the size of each supercell is defined by the distance of two adjacent 2*π* phase-fronts. For the optimization, we created a library formed by supercells with a single pillar with variable size and position. The combination of empty space (the equivalent of zero phase-delay) and a single pillar was in fact sufficient to implement the phase profile needed to deflect light in the respective orders.

We optimized for maximum power in the first order, while keeping its phase constant for all supercells (Fig. 3b). The final design showed more than 90% feedback efficiency (See Supplementary Information). With *λ* = 683 nm, *U* = 300 nm, *f* = 5 mm, *α*_x_ = 5° and *α*_y_ = 10°, the resulting metasurface has a rectangular shape of dimensions 0.8 mm × 1.5 mm. We move the metasurface using manual stages to tune the laser wavelength across the full operation range of the laser diode (Fig. 3d). We observe an increasing lasing threshold current when deviating from the central wavelength (683 nm).

The second prototype demonstrates how a stable collimated output can be achieved (Fig. 3e). To do so the supercell size was doubled, redefining the supercell as the union of two adjacent original supercells. This redefinition does not change the physics of the system, but formally the order of the retroreflected beam is now *N*_a_ = 2, while the *N*_a_ = 1 order is orthogonal to the metasurface and *N*_a_ = 0 represents the specular reflection. The intensity of light on the first order is zero if the two original supercells are identical, but any difference in the two will cause light to be coupled to the first order. By breaking the symmetry of two adjacent supercells (detailed description in the Supplementary Information) and taking into account the angle of incidence at each supercell position, we optimize the supercells to distribute 40% of the power into the feedback beam and to minimize the 0th order.

Here, $a\left(x,y\right)$ and $b\left(x,y\right)$ were chosen such that ${N}_{{\rm{b}}}\,=\,0$, ${N}_{{\rm{a}}}\in \left[{\mathrm{0,1,2}}\right]$ and for the second order the angles of incident and scattered light satisfy *α*_out_ = *α*_in_ on every position on the SCMS. Then, following laws of reflection and refraction, we have (Fig. 3e):

- For the zeroth order (*N*_a_ = 0, *N*_b_ = 0): *α*_out_ = −*α*_in_
- For the first order (*N*_a_ = 1, *N*_b_ = 0): *α*_out_ = 0
- For the second order (*N*_a_ = 2, *N*_b_ = 0): *α*_out_ = *α*_in_

Because the first order is orthogonal to the metasurface and has the same phase at each point, it is effectively a collimated beam.

We therefore implemented the focusing phase profile in Eq. 5 on the second instead of the first order. This corresponds to a division of *a*(*x, y*) in Eq. 7 by a factor of 2. The new supercell library range *S* ∈ [800 nm, 1400 nm], *U* = 300 nm is now doubled in comparison to the first prototype, as previously discussed. Figure 3g shows a schematic of the supercells sampling the required phase profile in the second order. As the second order deflection corresponds to a 4*π* phase ramp over the size of the supercell size, the size of each supercell is now chosen to have a dimension of the distance between two 2*π* phase fronts. Both focusing of the feedback beam and the creation of the collimated MECL output are controlled using the freedom of the curvilinear lattice, and again all the supercells must have the same phases of ${C}_{\mathrm{2,0}}\left(n,m\right)$ in their respective orders to constructively interfere.

To maximize its overlap with the diode output beam, we designed an elliptically shaped metasurface with *f* = 4 mm and a dimension of 5.8 mm and 2.5 mm along its major and minor axis, respectively.

The measured laser output is collimated to about 1 m and the wavelength can be tuned across the entire operating range of the laser diode (Fig. 3h). The device can achieve a wavelength resolution of less than 0.1 nm (limited only by the low frequency vibrations in the setup) by using piezoelectric stages (Fig. 3i). The emitted power (monitored via the photocurrent of the integrated photodiode as well as via a power meter measurement on the first order) changes drastically above laser threshold (Fig. 3j) and exceeds 10 mW. Figure 3k, l show the optical and the SEM picture of the metasurface.

### Laser with free-form holographic output

**Fig. 4:**
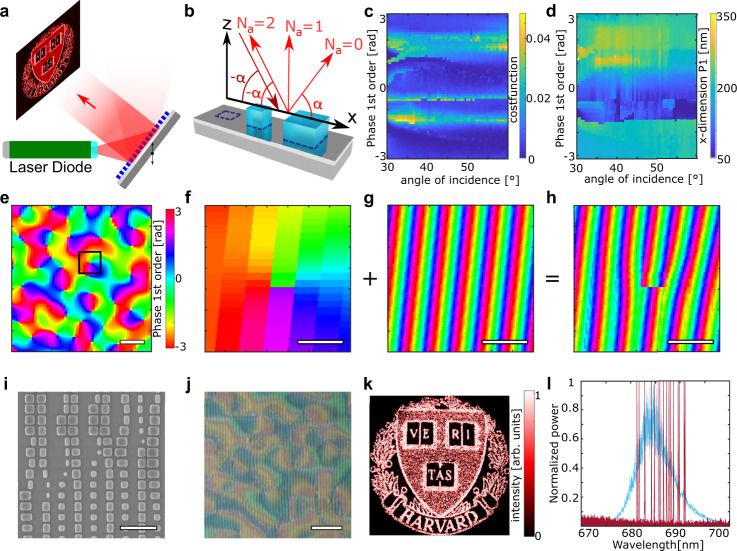
Metasurface external cavity laser (MECL) with holographic output beam. **a** Schematic of the MECL, showing the target holographic image. **b** Schematic of a supercell containing three pillar locations, one of them empty. The size of the supercell is chosen in such a way that the angle of the second local diffraction order matches the incident angle of the incoming light. **c** Cost function of the supercell library elements, see main text for definition. **d** Example of supercell-library parameter: *x*-dimension of the center pillar in each supercell of the library. **e** Detail of the designed holographic phase profile *C*_1,0_(*n, m*) imposed onto the first order, scale bar 10 µm. **f** Zoom in of the region marked by black rectangle in **e**, scale bar 2 µm. **g** For the same region in **f**, the profile of *G*_1,0_(*x, y*) is shown; scale bar 2 µm. **h** For the same region in F and G, the resulting phase profile in the first order *T*_1,0_(*n, m*), is shown; scale bar 2 µm. **i** SEM image of the metasurface, scale bar 2 µm. **j** Optical microscope image of the metasurface, scale bar 20 µm. **k** Hologram projected on and measured by a CCD camera. The Harvard logo occupies 16° of arc. **l** Wavelength tuning (red) and electroluminescence spectrum (blue) for *I* = 65 mA, having a central wavelength of 685 nm.

Finally, we demonstrated a device which generates an arbitrary holographic output, without affecting the light which is focused back to the laser facet (Fig. 4a). This also serves as a demonstration of the independent phase control on each diffraction order which is possible with supercell-metasurfaces.

We are using the coordinate system and supercell sizes of the second prototype, but with an extended library (see Supplementary Information). The feedback beam and the holographic output still correspond to the second and first order, respectively. As in the previous designs, the supercell library is designed to keep the same phase on the second order for all supercells so that the feedback beam is adequately focused on the laser facet. However, we now also optimize the phase of the first order to get library elements covering all phases needed for the desired hologram in the first order direction. The supercells contain two pillars (Fig. 4b) and were optimized to send 66 and 33% of the power into the second and first order, respectively. Figure 4c, d are a graphical representation of the cost function *f* (defined by Eq. 3) and an example of a supercell parameter of each supercell library element, respectively. The hologram phase profile was created using the Gerchberg–Saxton algorithm (GS, see Supplementary Information) and implemented onto the first order (Fig. 4e). The phase maps obtained with the GS algorithm often contain topological singularities, which are non-removable discontinuities formed by a single point where the phase is not defined and surrounded by every possible value of the phase. Many of these singularities are visible in Fig. 4e. Figure 4f shows a zoomed-in region around one of these singularities (marked by a black square in Fig. 4e). Figure 4f–h show a pictorial representation of Eq. 2 for this case: the discrete holographic phase profile (Fig. 4f) corresponds to the phase of ${C}_{\mathrm{1,0}}\left(n,m\right)$ and is added to the phase of ${G}_{\mathrm{1,0}}\left(x,y\right)$ (see Eqs. 1 and 2), which has a uniform phase gradient (Fig. 4g). Figure 4h then shows the final phase profile implemented on the first order of the supercell metasurface and creating the hologram, showing a characteristic fork pattern^31^. The structure of the hologram can be seen both in the SEM (Fig. 4i) and the optical image (Fig. 4j) of the fabricated supercell metasurface. The achieved hologram (Fig. 4k) has an angular size of 16° of arc once projected and can be wavelength-tuned across the entire operating range of the laser diode (Fig. 4i). This prototype demonstrates the power of the multifunctional supercell approach, creating a feedback beam on one order and an arbitrary hologram or beam profile on another, while maintaining the wavelength tuneability.

We demonstrated a generalization of metasurfaces using supercells harnessing non-locality, where independent functions are implemented on each order overcoming the shortcomings of the commonly used design approaches. Existing metasurfaces and metagrating implementations are special cases of this general type, and our results can be extended including polarization functions and dispersion engineering. While our experimental realizations are based on simple supercells containing up to two rectangular pillars, combining our approach with free form, inverse-designed nanostructures will enable the realization of metasurfaces with unprecedented capabilities. For instance, the creation of efficient wide-angle holograms in far field can be achieved by splitting the far field radiation pattern of the hologram into smaller portions having a smaller solid angle. Then, each portion can be implemented independently by a different diffraction order. That way, multiple diffraction orders can work synergistically to implement a single wide hologram. Our external cavity laser concept with arbitrary beam control capabilities cannot be realized by existing metasurface and metagrating implementations. The concept potentially outperforms standard ECL, paving the way to new active optoelectronic devices. Lasers based on other gain media and light emitting systems can be controlled with the same method.

## Methods

### Sample fabrication

The supercell-metasurfaces were fabricated following a combination of the processes reported in refs. ^11,12,17^. To create the epitaxial silver layer, we used a polished silicon wafer cut along the <111> crystal face as substrate. The wafer was cleaned in a 3:1 mixture of hot H_2_SO_4_ and H_2_O_2_, rinsed in water, cleaned in HF 49% to remove any native oxide residue. Ag was then sputtered^12,17^ forming a monocrystalline epitaxial film. A protective layer of 10 nm of Al_2_O_3_ was deposited with atomic layer deposition (ALD). To define the pillars, ZEP resist was spin coated, exposed with electron beam lithography and developed with cold oxylene. The standard metasurface process^11^ was then used to create the pillars (See Supplementary Information for a detailed process flow).

### Numerical simulations

The supercells optimization was performed using the rigorous coupled wave analysis solvers Reticolo and S4^37,46^, assuming *n*_Ag_ = 0.061 + 4.673i, *n*_SiO2_ = 1.457, and *n*_TiO2_ = 2.346 (See Supplementary Information for a detailed process flow).

### Measurements

Helical and Bessel beam of the first presented supercell metasurface were imaged using a microscope arm that included a 100× objective (NA 0.9), a lens with a focal distance of 15 mm and a CCD camera with a 1280 × 1024 Pixels resolution. The zeroth order was imaged in the far-field using a lens with focal distance of 5 mm and the same CCD camera. A beam of reduced diameter was used to ensure that the gaussian tail of the incident beam vanishes at the edge of the metasurface (approx. 250 µm, see Supplementary Information for details). The power of each order was measured using a power meter and was then compared to the laser output power. To measure the MECL, we used piezoelectric elements with a resolution of 20 nm to control the metasurface position. The MECL output was coupled into a spectrometer with theoretical resolution of 0.06 nm (grating with 1200 lines/mm). The power was determined using a power meter. For measurement details see Supplementary Information.

## Supplementary information

Supplementary Information

## Data Availability

All data supporting the findings of this study are available within the article and its Supplementary Information files and from the corresponding authors upon reasonable request.

## Code availability

All code will be provided upon request to the authors. Please address your requests for data and code to the corresponding authors.

## References

[1] Rubin NA (2019). Matrix Fourier optics enables a compact full-Stokes polarization camera. Science.

[2] Silva A (2014). Performing mathematical operations with metamaterials. Science.

[3] Kildishev AV, Boltasseva A, Shalaev VM (2013). Planar photonics with metasurfaces. Science.

[4] Chen WT, Zhu AY, Capasso F (2020). Flat optics with dispersion-engineered metasurfaces. Nat. Rev. Mater..

[5] Chen HT, Taylor AJ, Yu N (2016). A review of metasurfaces: physics and applications. Rep. Prog. Phys..

[6] Shaltout AM (2019). Spatiotemporal light control with frequency-gradient metasurfaces. Science.

[7] Ni X, Kildishev AV, Shalaev VM (2013). Metasurface holograms for visible light. Nat. Commun..

[8] Glybovski SB, Tretyakov SA, Belov PA, Kivshar YS, Simovski CR (2016). Metasurfaces: from microwaves to visible. Phys. Rep..

[9] Shaltout AM, Shalaev VM, Brongersma ML (2019). Spatiotemporal light control with active metasurfaces. Science.

[10] Overvig AC, Malek SC, Yu N (2020). Multifunctional Nonlocal Metasurfaces. Phys. Rev. Lett..

[11] Shi Z (2018). Single-layer metasurface with controllable multiwavelength functions. Nano Lett..

[12] Shi Z (2020). Continuous angle-tunable birefringence with freeform metasurfaces for arbitrary polarization conversion. Sci. Adv..

[13] Kamali SM (2017). Angle-multiplexed metasurfaces: encoding independent wavefronts in a single metasurface under different illumination angles. Phys. Rev. X.

[14] Song Q (2020). Ptychography retrieval of fully polarized holograms from geometric-phase metasurfaces. Nat. Commun..

[15] Wang, D. et al. High-efficiency metadevices for bifunctional generations of vectorial optical fields. *Nanophotonics* **1**, 685–695 (2020).

[16] Byrnes SJ, Lenef A, Aieta F, Capasso F (2016). Designing large, high-efficiency, high-numerical-aperture, transmissive meta-lenses for visible light. Opt. Express.

[17] Ding F, Wang Z, He S, Shalaev VM, Kildishev AV (2015). Broadband high-efficiency half-wave plate: a supercell-based plasmonic metasurface approach. ACS Nano.

[18] Yeung c (2021). Multiplexed supercell metasurface design and optimization with tandem residual networks. Nanophotonics.

[19] High AA (2015). Visible-frequency hyperbolic metasurface. Nature.

[20] Moccia M (2017). Coding metasurfaces for diffuse scattering: scaling laws, bounds, and suboptimal design. Adv. Opt. Mater..

[21] Malek SC, Overvig AC, Shrestha S, Yu N (2020). Active nonlocal metasurfaces. Nanophotonics.

[22] Yermakov O (2020). Nanostructure-empowered efficient coupling of light into optical fibers at extraordinarily large angles. ACS Photonics.

[23] Arbabi A (2020). Increasing efficiency of high numerical aperture metasurfaces using the grating averaging technique. Sci. Rep..

[24] Phan T (2019). High-efficiency, large-area, topology-optimized metasurfaces. Light Sci. Appl..

[25] Jiang, J., Phan, T. & Fan, J. A. High efficiency aperiodic metasurfaces based on variable phase shifting element spacing. in *Optics InfoBase Conference Papers* vol. Part F93-C FF3C.1 (Optical Society of America, 2018).

[26] Jiang J (2019). Free-form diffractive metagrating design based on generative adversarial networks. ACS Nano.

[27] Paniagua-Domínguez R (2018). A metalens with a near-unity numerical aperture. Nano Lett..

[28] Neder V, Ra’Di Y, Alù A, Polman A (2019). Combined metagratings for efficient broad-angle scattering metasurface. ACS Photonics.

[29] Gao S, Lee SS, Kim ES, Choi DY (2018). Vertically integrated visible and near-infrared metasurfaces enabling an ultra-broadband and highly angle-resolved anomalous reflection. Nanoscale.

[30] Sroor H (2020). High-purity orbital angular momentum states from a visible metasurface laser. Nat. Photonics.

[31] Yu N (2011). Light propagation with phase discontinuities: generalized laws of reflection and refraction. Science.

[32] Aieta F, Kats MA, Genevet P, Khorasaninejad R, Capasso F (2015). Achromatic metasurfaces by dispersive phase compensation. 2015 IEEE Photonics Conference.

[33] Ren H (2019). Metasurface orbital angular momentum holography. Nat. Commun..

[34] Bliokh KY, Dressel J, Nori F (2014). Conservation of the spin and orbital angular momenta in electromagnetism. N. J. Phys..

[35] Rubinsztein-Dunlop H (2016). Roadmap on structured light. J. Opt..

[36] Allen L, Padgett MJ, Babiker M (1999). The orbital angular momentum of light. Prog. Opt..

[37] Yang J, Fan JA (2017). Topology-optimized metasurfaces: impact of initial geometric layout. Opt. Lett..

[38] Liu V, Fan S (2012). S4: a free electromagnetic solver for layered periodic structures. Comput. Phys. Commun..

[39] Hugonin, J. P. & Lalanne, P. Reticolo software for grating analysis. Preprint at *arXiv* (2021).

[40] Fan JA (2020). Freeform metasurface design based on topology optimization. MRS Bull..

[41] Sell D, Yang J, Doshay S, Yang R, Fan JA (2017). Large-angle, multifunctional metagratings based on freeform multimode geometries. Nano Lett..

[42] Elsawy MMR, Lanteri S, Duvigneau R, Fan JA, Genevet P (2020). Numerical optimization methods for metasurfaces. Laser Photonics Rev..

[43] Xie YY (2019). Vertical cavity metasurface-emitting lasers (VCMELs) for programmable directional lasing emissions. Nat. Nanotechnol..

[44] Xu L (2015). Metasurface external cavity laser. Appl. Phys. Lett..

[45] Kim, A. D. et al. Cavity length dependence of a metasurface quantum-cascade laser with electrically switchable polarization. In *Optics and Photonics for Sensing the Environment* EW4H-EW44 (OSA Publishing, 2020).

[46] Zhu H, Patnaika S, Walsh TF, Jared BH, Semperlotti F (2020). Nonlocal elastic metasurfaces: enabling broadband wave control via intentional nonlocality. Proc. Natl Acad. Sci. USA.

[47] Zhu AY (2019). Compact aberration-corrected spectrometers in the visible using dispersion-tailored metasurfaces. Adv. Opt. Mater..

[48] Khorasaninejad M, Chen WT, Oh J, Capasso F (2016). Super-dispersive off-axis meta-lenses for compact high resolution spectroscopy. Nano Lett..

[49] Mroziewicz B (2008). External cavity wavelength tunable semiconductor lasers—a review. OptoElectron. Rev..

