# Supplementary material for: Multifunctional wide-angle optics and lasing based on supercell metasurfaces
Source: Nat Commun. 2021 Jun 18;12:3787. doi: 10.1038/s41467-021-24071-2 (PMC8213840; doi:10.1038/s41467-021-24071-2)
Supplement: Supplementary file 1 — Supplementary Information [file 41467_2021_24071_MOESM1_ESM.pdf]

Supplementary Information for Multifunctional wide-angle optics and lasing based on supercell metasurfaces

Christina Spägele,[1] Michele Tamagnone,[1,2*], Dmitry Kazakov[1], Marcus Ossiander [1], Marco Piccardo[1,3], Federico Capasso[1*]

[1]Harvard John A. Paulson School of Engineering and Applied Sciences, Harvard University, Cambridge, Massachusetts 02138, USA
[2]Istituto Italiano di Tecnologia, via Morego 30, 16163 Genova, Italy
[3]CNST – Fondazione Istituto Italiano di Tecnologia, Via Giovanni Pascoli, Milan, Italy

*To whom correspondence should be addressed;
E-mail: mtamagnone@seas.harvard.edu, capasso@seas.harvard.edu.

Supplementary Note 1: Efficiency limits of spatially multiplexed (interleaved) metasurfaces

As shown in Supplementary Figure 1A, interleaved metasurfaces can implement multiple functions at various angles, but they have important efficiency limitation. If N separate functions at different angles are implemented, each carrying an equal fraction of the original incident power, then in the local limit the overall useful efficiency of the device drops to approximately $1/N$. The reason is that the field contributions to each function are divided by a factor N (easily seen replacing the metasurface elements by equivalent radiators and using linearity of Maxwell's equations). However, the power is reduced by the square of that amount, i.e. N^2. The overall efficiency is then $N \cdot (1/N^2) = 1/N$. The remaining fraction $(N-1)/N$ of the total power is radiated in spurious diffraction orders which appear due to the interleaved rows/columns. These orders may be evanescent, in that case the power of these evanescent waves is randomly scattered in one of the orders or backwards with respect to the incident light.

Supplementary Note 2: Mathematical framework

The effective supercell-metasurface complex transmission profile $T_{\mathrm{N_a,N_b}}(x,y)$ associated to the grating order $(N_\mathrm{a}, N_\mathrm{b})$ can be described as

$$T_{\mathrm{N_a,N_b}}(x,y) = C_{\mathrm{N_a,N_b}}(n,m)\,\underbrace{e^{2\pi i[N_\mathrm{a}a(x,y)+N_\mathrm{b}b(x,y)]}}_{G_{\mathrm{N_a,N_b}}(x,y)} \tag{1}$$

where N_a and N_b are the indices of the considered order, n and m are the integer indexes of the supercell closest to the position (x,y) and C is the complex transmission coefficient in that order and supercell. G represents the uniform phase gradient associated to the diffraction order. The expression for G is quantized in $N_\mathrm{a}, N_\mathrm{b}$ due to its periodic nature over the supercell. The product of the two complex amplitudes causes the phases to be added as shown graphically in Supplementary Figure 2A), which shows the phases of four rectangular supercells at the corner of a linear lattice on their respective local orders (k,l).

The lattice on which the supercells of a supercell-metasurface are positioned on is not bound to be linear (i.e. a Bravais lattice generated by two lattice vectors **a** and **b**), but can be described in a more general way by two continuous, real-valued functions $a(\mathbf{r})$ and $b(\mathbf{r})$, where $\mathbf{r} = (x,y)$ is the position vector on the substrate plane. The functions are defined so that the lattice points occur at positions **r** such that both $a(\mathbf{r})$ and $b(\mathbf{r})$ are integer numbers (n and m respectively). Then the condition $a(\mathbf{r}) = n \in Z$ is satisfied by a family of curves (in red in the example in Supplementary Figure 2B), where each value of n generates a separate curve. Similarly, the condition $b(\mathbf{r}) = m \in Z$ is satisfied by the blue family of curves. The intersection points are the lattice points, satisfying both conditions.

The functions $a(\mathbf{r}) = a(x,y)$ and $b(\mathbf{r}) = b(x,y)$ can be interpreted as a system of *curvilinear coordinates* and we assume that they are sufficiently smooth so that the lattice can be approximated *locally* as parallelograms in a Bravais lattice. Within this formalism, the local lattice vectors are obtained from the partial derivatives in a and b as $\mathbf{a} = \partial_\mathrm{a}\mathbf{r}$ and $\mathbf{b} = \partial_\mathrm{b}\mathbf{r}$. The lattice can be chosen in a variety of ways, and changing the distance of the curves one can control the size of the supercells and hence the number and direction of their local orders.

It is important to note that, equivalent to the practice of typical simulation environments like Reticolo, the presented order dependent transmission amplitudes are not describing the change in amplitude of the electric field, but of the normal component of the pointing vector's real part, as the latter is conserved even for transmitted orders with a non-zero deflection angle. Its phase, on the other hand, is equivalent to the phase of the electric field, as the phase of the electric field is not affected by a change of propagation direction. The concept of replacing the fields' amplitude with the real part of the Poynting vector is also known as powerwaves. With this definition we can ensure, that the overall transmission/reflection coefficients sum up to 1 in case of a lossless metasurface of 100% efficiency.

Only for the zeroth order($N_a = 0, N_b = 0$), neglecting evanescent waves, the complex coefficient $T_{0,0}(x,y) = C_{0,0}(n,m)$ also describes the delay and attenuation of both the electric

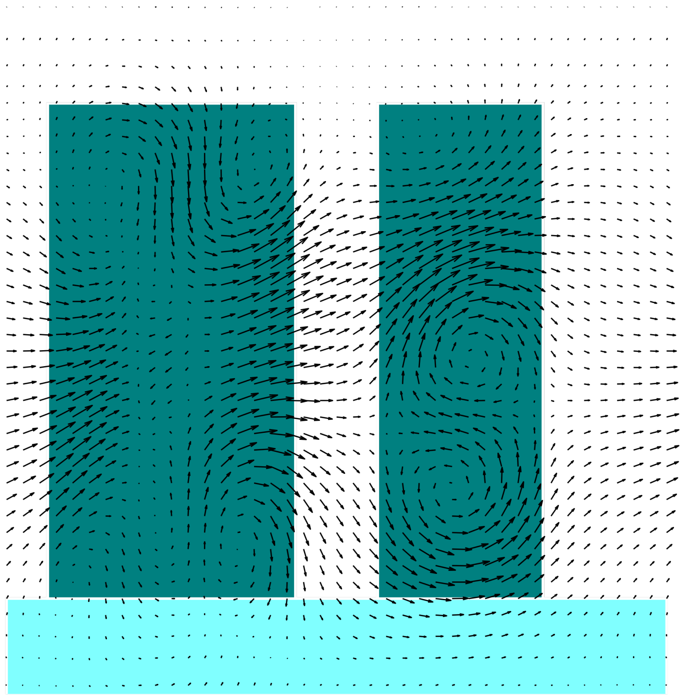

Supplementary Figure 1: **Example of Poynting vector in a supercell.** The plot reveals that a flow of optical power exists between the pillars in the supercell, proving that the existence of non-local interactions. For the presented simulation, two square pillars (n=2.3) of side-length 0.2μm and 0.3μm, respectively, were positioned on a glass substrate (n=1.45). Both pillars have a height of 0.6μm. The simulated supercell has a dimension of 0.8μm x 0.4μm with Bloch boundary conditions in the xy direction. The Poynting vector presented in the figure is determined on the plane crossing the center of the two pillars (y=0). The incident s-polarized plane wave is tilted by an angle of 30°.

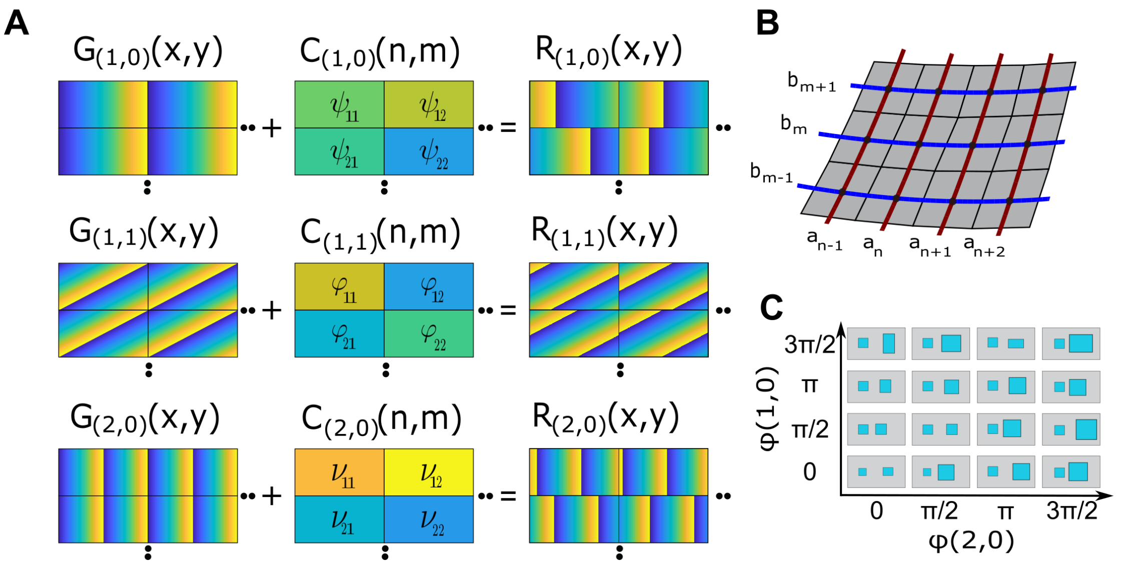

Supplementary Figure 2: **The supercell-metasurface framework** (**A**) Pictorial representation of the SCMS framework, showing how it can implement independent functionalities in different directions while handling large angles of deflections. (**B**) The curvilinear lattice is defined by two sets of curves a and b, marked in blue and red. The intersection of the lines a_{n} and b_{m} mark the position of supercell (n, m). Changing its dimension controls the grating period and hence the number and angle of the local diffraction orders. (**C**) Schematic of a supercell library, connecting the inlay of the supercell with their order dependent phases.

and magnetic fields at a given position (x, y) caused by cell (n, m), as the wave passes the supercell without being deflected.

For higher orders, the situation is more complex, as the beam is diffracted by the supercell, which locally behaves as a diffraction grating. As the angle of the diffracted beam is different from the incident one, the beam diameter changes upon deflection and the electric field is not conserved even for a deflection efficiency of 100%. $T_{N_a,N_b}(x, y)$ then describes the ratio of the normal components of the respective Poyntings vector's real parts, which are conserved under a deflection with efficiency of 100%.

By decomposing the transmitted field as the sum of a finite number of plane waves (the diffracted orders), the phase of each beam is described by the coefficient C_{N_a,N_b}. This observation is based on the plane wave decomposition applied to a Bloch-periodic environment, and is independent by the particular physical system considered (e.g. it can be applied also to acoustic metasurfaces).

The generalization to curvilinear lattices is achieved by replacing the phase gradient of a linear uniform grating $e^{2\pi i[N_a(x/L)+N_b(y/H)]}$ with the curvilinear profile, $e^{2\pi i[N_a a(x,y)+N_b b(x,y)]}$ where the integer part of the functions $a(x, y)$ and $b(x, y)$ represent the indices of the cell, and the fractional part is the position of the point within the cell. They have therefore the exact same role of the terms x/L and y/H in the uniform grating.

Supplementary Note 3: Numerical simulations and supercell-libraries

3.1. Device 0: SCMS demonstrator in transmission

As base for the metasurface/metagratings we create a library of locally similar grating elements obeying the following protocol: On the single-element-level, we optimize the geometrical properties of a grating element (height 600nm) intended to achieve a set of goal optical grating properties (phase adherence, efficiency, output/feedback ratio) using a hill-climb algorithm with annealing to escape shallow local minima. Optical properties of grating elements were simulated for a periodic array of identical elements using the Fourier Modal Method implemented by S4 (http://dx.doi.org/10.1016/j.cpc.2012.04.026). We optimize elements (consisting of two TiO_2 pillars on a Si substrate) towards full phase coverage in order 1 and 2 for the laser wavelength 638nm, while equally distributing the powers between the three orders (Supplementary Figure 3). The substrate was included in the simulation to correct for substrate effects. To retain local similarity, we use the geometrical properties of already available elements with similar optical properties as starting point of our optimization. If elements do not display satisfying optical properties after the initial optimization we re-run the optimization after more results in the optical vicinity of the disappointing elements become available.

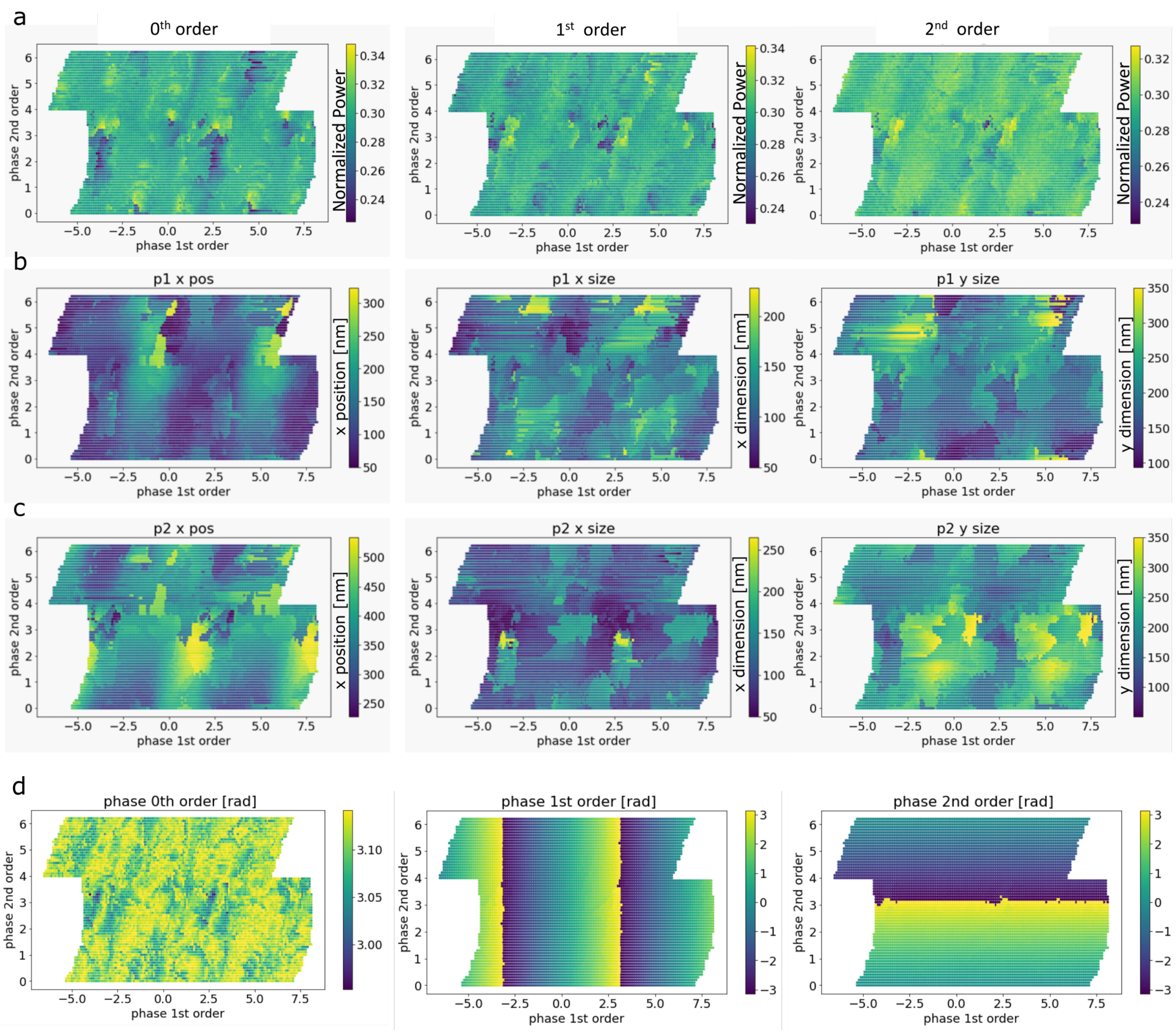

Supplementary Figure 3: **Supercell library for the SCMS demonstrator in transmission.** The presented supercell library has a full 2π coverage in the first and second order, while the zeroth order phase is kept constant. Each library-element is represented as one point in the graph - its color indicates: (**A**) The simulated normalized power of each order. (**B**) The dimension and x-position of pillar 1. (**C**) The dimension and x-position of pillar 2. (**D**) The simulated phase of each order.

3.2. Device 1: TM polarized feedback test

The device functions as a proof of concept of the metasurface external cavity laser and was designed to send 100% of the incident light back to the laser facet. It was sufficient to choose a supercell of two rectangular pillars, one of which empty. While height (300nm) and y-position of the pillar (center) were fixed, the parameters x-dimension, y-dimension and position in x were optimized using a gradient-descent optimization (Supplementary Figure 4). To retain local similarity, we use the geometrical properties of already available elements with similar optical properties as starting point of our optimization. As the first prototype was designed as a proof of concept, the library consisted of 29 different supercells.

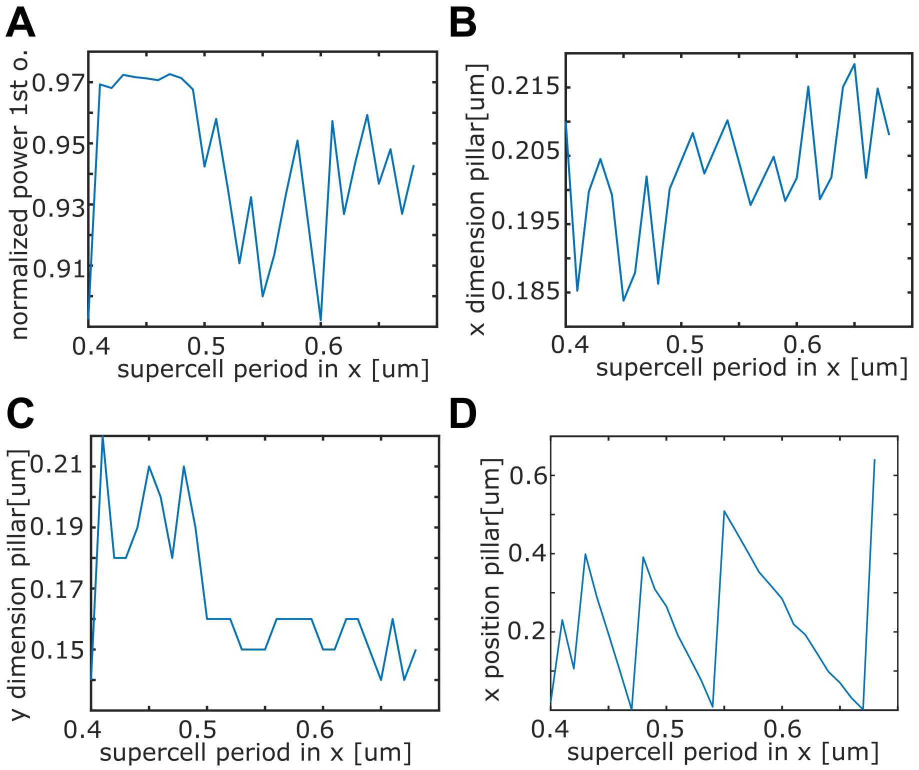

Supplementary Figure 4: **Supercell library for 1st prototype of MECL.**(**A**)normalized power of first order of the library elements. (**B**)x-dimension of pillar for all library elements. (**C**)y-dimension of pillar for all library elements. (**D**)x-position of pillar for all library elements.

3.3. Device 2: TE polarized collimated output

Transitioning from the first to the second prototype, a rotation of the laser cavity switched the diverging angles to $\alpha_x = 12°$ and $\alpha_y = 6°$ as well as the impinging polarization on the supercells from p to s. The collimated beam was designed using a TE polarized configuration,

because it was found empirically that with the TE configuration the designed library was much simpler and the behavior was naturally broadband.

The first step was to optimize a unit cell similar to the first demonstrator, sending 100% of the incident light back to the laser facet. The supercell uses one square pillar (120nm tall) placed in the center of the supercell and with the size optimized so that the zeroth order vanishes and all the power is sent to the facet of the laser.

The second step consists in defining a doubled supercell, formed by two of the previosuly optimized supercells adjacent along the x direction. This redefinition does not change the physics of the system, although the orders are renumbered by this definition and the feedback is now along order 2, while order 1 is orthogonal to the supercell-metasurface (acting as the output beam as discussed). Because the two halves of the supercell are identical, no power is diffracted in the first order. However, any difference created in the two halves will scatter light in it. This can be achieved by either moving the pillar on the left to the left and the pillar to the right to the right (creating an asymmetry) or by making one pillar slightly larger and the other slightly smaller. The first method is used in this case, to achieve a ratio between the feedback and the output beam of 1:1.3 (equivalent to feedback of about 40%) and having most of the light scattered in the output beam. The simulations were performed with Reticolo.

3.4. Device 3: TE polarized holographic output

As base for the SCMS we create a library of locally similar grating elements obeying the following protocol: On the single-element-level, we optimize the geometrical properties of a grating element (height 120nm) intended to achieve a set of goal optical grating properties (phase adherence, efficiency, output/feedback ratio) using a hill-climb algorithm with annealing to escape shallow local minima. Optical properties of grating elements were simulated for a periodic array of identical elements using the Fourier Modal Method implemented by S4 (http://dx.doi.org/10.1016/j.cpc.2012.04.026). To obtain a library with full phase coverage in order 1, we optimize a set of grating elements to achieve all phase combination for orders 1 and 2 for the design's intended central angle-of-incidence and output-feedback ratio. From this set, we pick the phase of order 2 achieving the best combined merit for all order 1 phases and fix it for the subsequent optimization. We then, for all angles-of-incidence, optimize elements towards full phase coverage in order 2. To retain local similarity, we use the geometrical properties of already available elements with similar optical properties as starting point of our optimization. If elements do not display satisfying optical properties after the initial optimization we re-run the optimization after more results in the optical vicinity of the disappointing elements become available.

Supplementary Note 4: Hologram generation with GS algorithm

The hologram was generated using a modified version of the Gerchberg–Saxton (GS) algorithm. The phase profile is first generated using this algorithm on a regular lattice made of tiles which

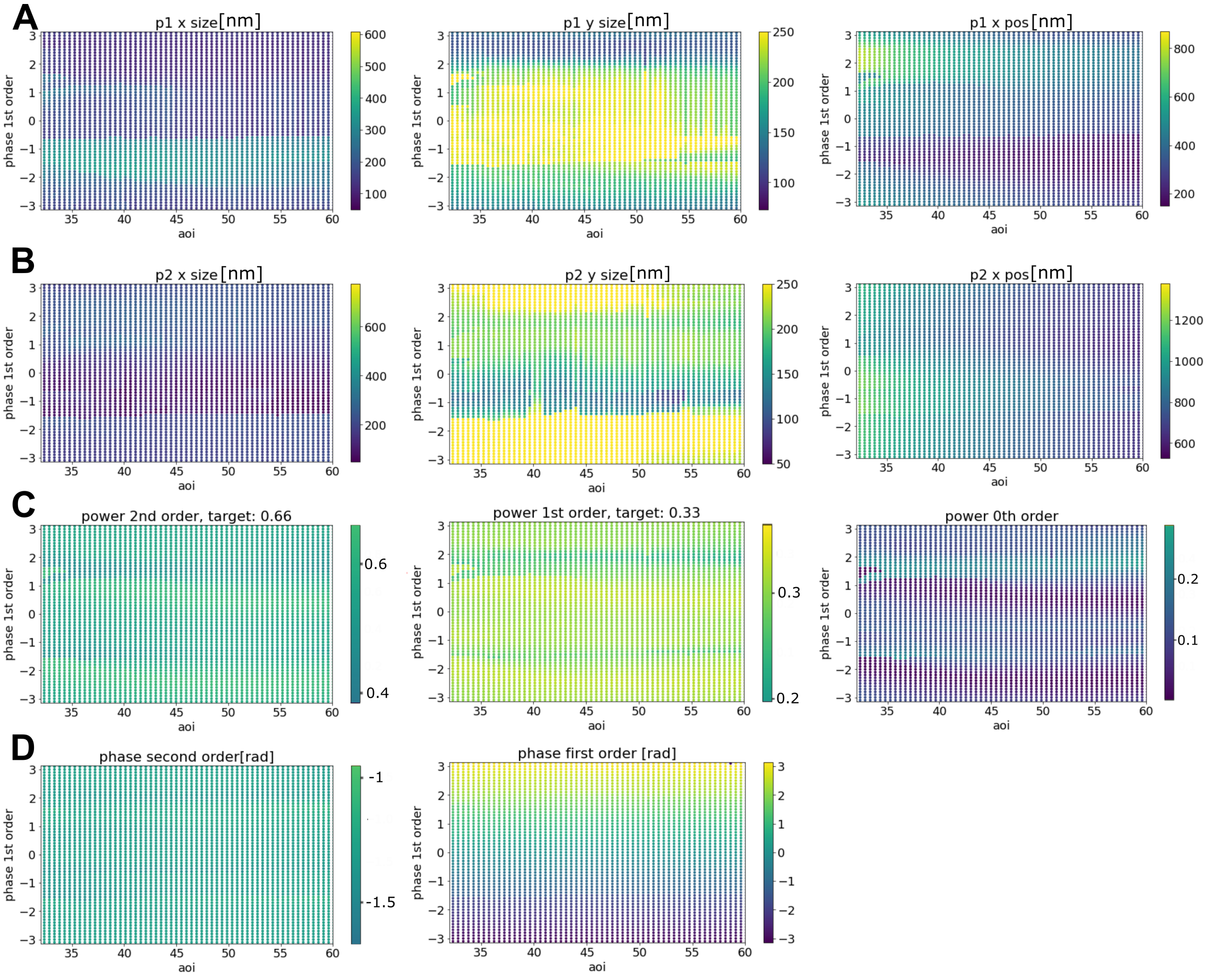

(**C**) order dependent simulated power (**D**) order dependent simulated phases

Supplementary Figure 5: **Supercell library for the MECL with holographic output.**(**A**) dimension and x-position of pillar 1. (**B**) Dimension and x-position of pillar 2.
(**C**) order dependent simulated power (**D**) order dependent simulated phases

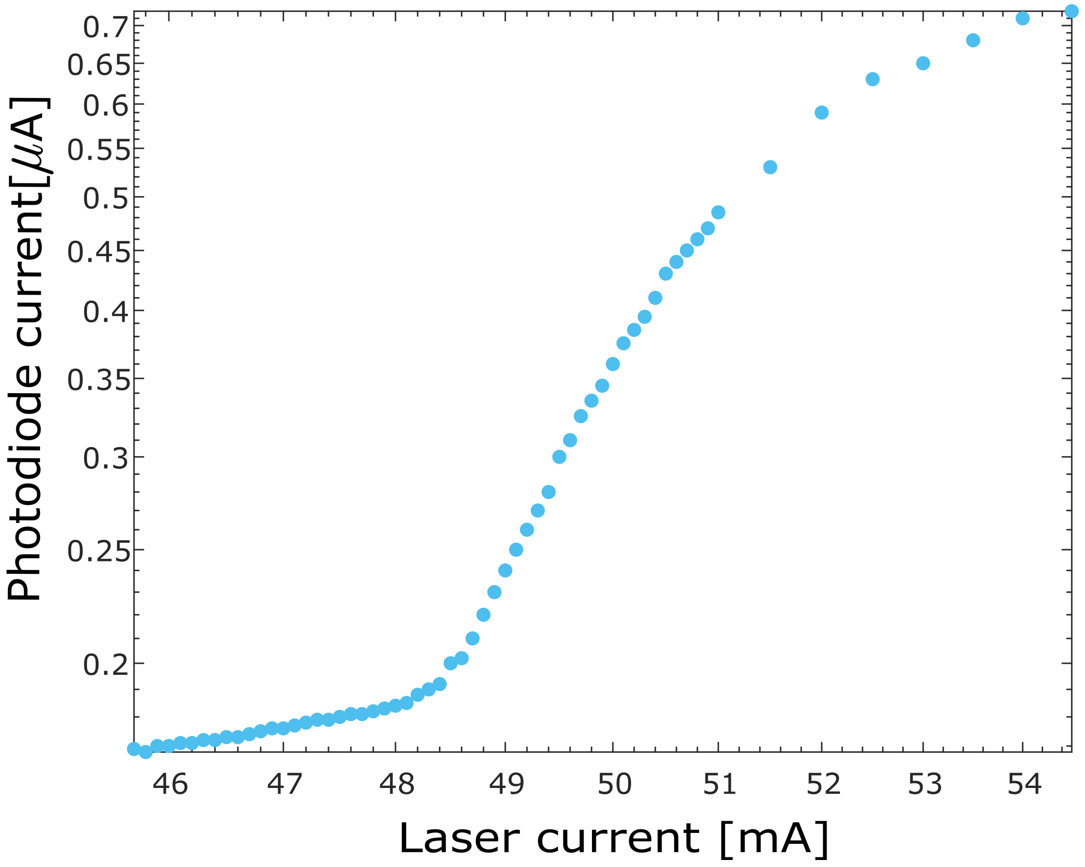

Supplementary Figure 6: **Characterization of the lasing threshold at central wavelength (log-log scale).**

are 300nm along the y direciton and 900nm along x. On the actual supercell-metasurface, the size along x is actually variable with position, so 900 nm was chosen because it corresponds to the width of the supercells in the center of the metasurface (for incidence angle of 45 degrees approximately). 100 iterations of the GS algorithm were used.

The actual phase profile on the supercell-metasurface cells was obtained from the one generated with the GS algorithm by resampling it with a two-dimensional interpolation. However, the phase cannot be interpolated directly since it is defined as modulo-2π. Therefore we used unitary modulus to calculate locally the real and imaginary parts. We interpolated those and then computed locally the phase from these interpolated quantities, to avoid artifacts. The metasurface was then designed choosing the supercells so that the phase on the 1st diffraction order would match as close as possible to the required phase computed with the interpolation.

Supplementary Figure 7 shows the original target image, the expected far field after running the hologram and the final experimentally measured results.

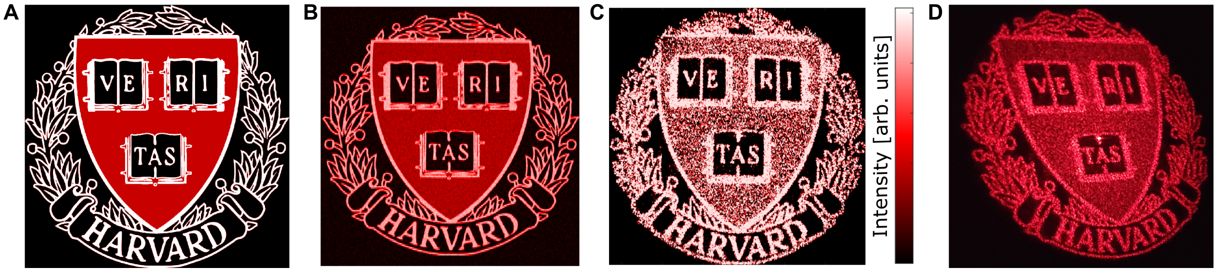

Supplementary Figure 7: **Hologram synhtesis with Gerchberg–Saxton algorithm.**(**A**) Original image used to create hologram. (**B**) Expected far field image after running the Gerchberg–Saxton algorithm. (**C**) Actual hologram imaged with the experimental setup. Due to the limited size of the CCD camera this picture was taken in three parts and stiched together. (**D**) optical image of the hologram projected on a tilted screen.

Supplementary Note 5: Detailed Fabrication Process

Supplementary Figure 8 summarizes the fabrication steps. To create the epitaxial silver layer we used a polished silicon wafer cut along the $< 111 >$ crystal face as substrate. The wafer was cleaned in a 3:1 mixture of hot H_2SO_4 and H_2O_2, rinsed in water, cleaned in HF 49% to remove any native oxide residue. Ag was then sputtered forming a monocrystalline epitaxial film. A protective layer of 10nm of Al_2O_3 was deposited with atomic layer deposition (ALD). To define the pillar, ZEP resist was spin coated, exposed with electron beam lithography and developed with cold oxylene. ALD TiO_2 was then deposited on the patterned resist, and the excess oxide was etched back with RIE using a fluorine based recipe. The resist was removed using Remover PG at 80 C and the sample was rinsed in acetone, IPA and cleaned using oxygen plasma.

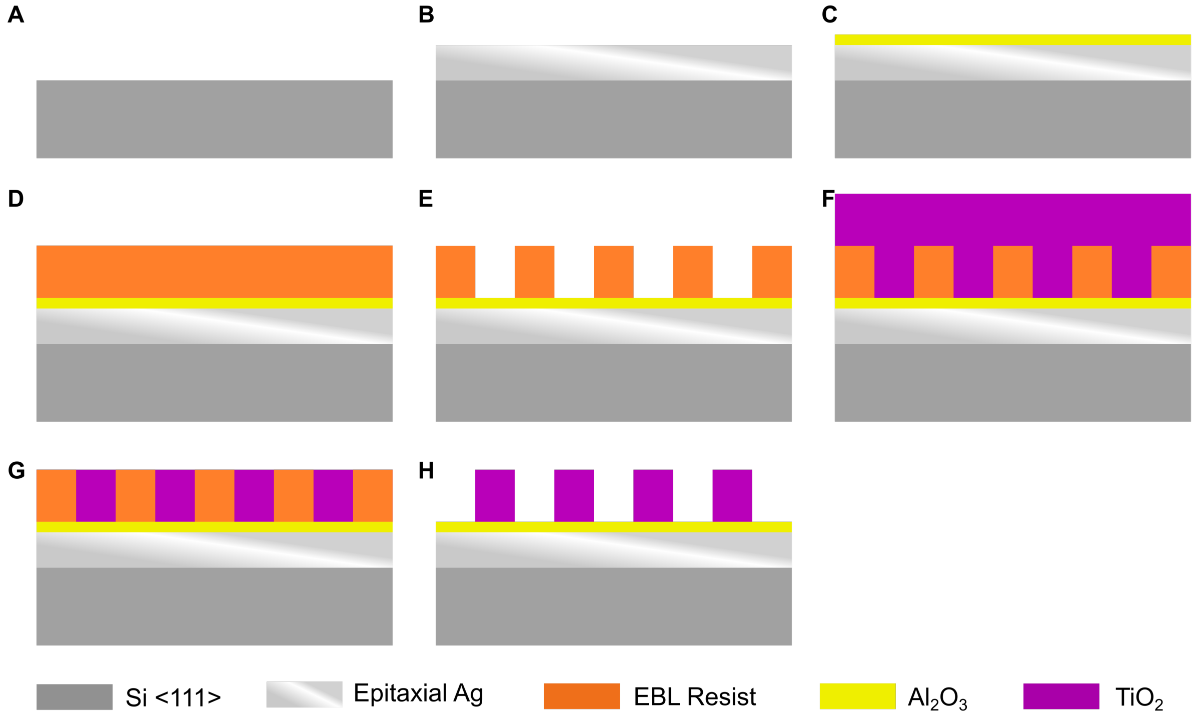

Supplementary Figure 8: **Fabrication process.** (**A**) Initial Si $< 111 >$ substrate cleaning. (**B**) Sputtering of epitaxial silver. (**C**) ALD deposition of Al_2O_3. (**D**) Spin coating e-beam resist. (**E**) e-beam exposure and development. (**F**) TiO_2 deposition using ALD. (**G**) RIE etch back. (**H**) Resist removal and final cleaning.

Supplementary Note 6: The measurement setup

6.1. Device 0 measurement

6.1.1 1st and 2nd order

As the Bessel beam has to be imaged in the proximity to the metasurface, it needs to be measured by an objective. We therefore also focused the 2nd order OAM beam, so that we can measure both orders with the same setup. The SCMS was positioned on a three-axis linear stage and illuminated by a collimated beam of a laser with wavelength 637nm. The 1st and 2nd order were then imaged using a microscope arm consisting of a 100x objective, a lens with a focal distance of 200mm and a CCD camera (sensor format 1/2”,resolution 1280 x 1024 Pixels). Supplementary Figure 10b,c are showing a schematic of the setup.

6.1.2 0th order

As the waist of our measurement beam exceeded the size of the metasurface, we narrowed the collimated beam by using a collimation lens of f=25.4mm and a 10x objective (Supplementary Figure 10a) and projected the zeroth order onto the same CCD camera using a lens of focal distance f=5cm. As there is no additional structuring on the zeroth order, a simple far field projection is suitable to observe the effect of the metasurface on the incident light.The CCD is placed in focal distance to the lens. The corresponding far field image is shown in the main paper and in Supplementary Figure 9a. Supplementary Figure 9b shows the far-field of the same beam when illuminating the glass substrate only.

6.1.3 Power measurement

The power of the respective order was measured with a powermeter and then compared to the total power of the laser diode output. The measured device showed a normalized power of 0.27, 0.21 and 0.30 on the 0th, 1st and 2nd order, respectively.

6.2. MECL measurement

The laser diode is mounted in a TEC cooled laser mount and positioned on a linear stage in y-direction (Supplementary Figure 11A). The supercell-metasurface was mounted at an angle of 45° with respect to the laser diode output and positioned on 3-axis stage. The stage enabled both fine adjustment (open loop piezoelectric element, minimal resolution 0.2nm) and coarse adjustment (manual micrometer screws). A mirror was glued onto the laser mount that reflects the output beam away from the mount. Optionally, the mirror can be placed on a separate support instead. For the measurement of the spectrum, the output was coupled into the fiber of a spectrometer (resolution 0.06nm), (Supplementary Figure 11 B). A plano-convex lens (f=4.5cm) was added to the setup when projecting the hologram onto a CCD-camera (monochromatic, sensor format 1/2”,resolution 1280 x 1024 Pixels) (Supplementary Figure 11 C)

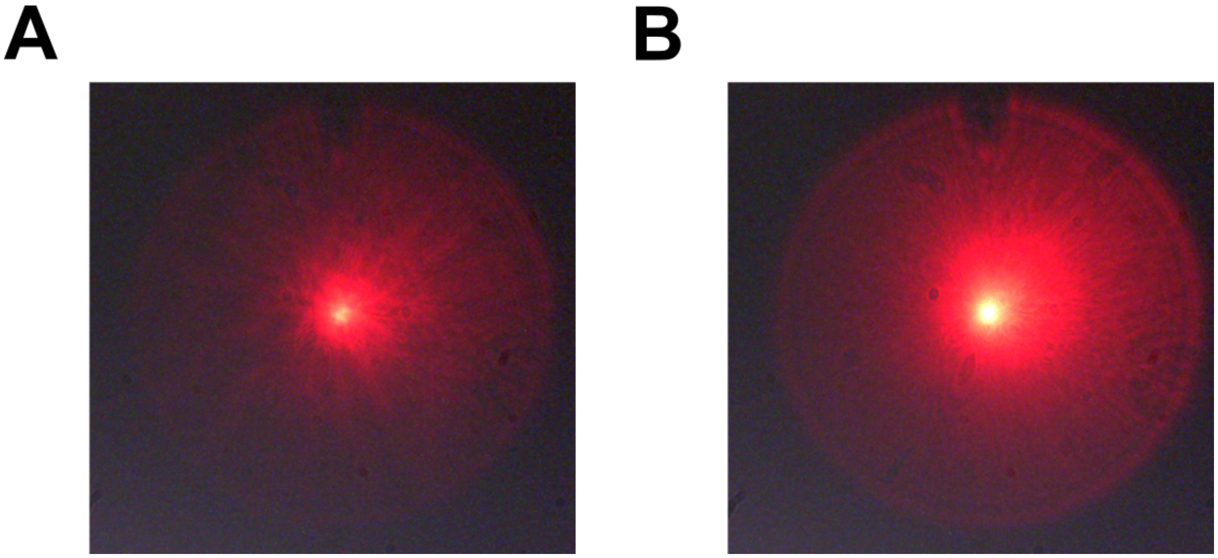

Supplementary Figure 9: **The MECL setup** (**A**) Farfield image of the zeroth order illumnated by a beam of approx 250um diameter (**B**) Farfield of the same beam when illuminating the glass substrate only.

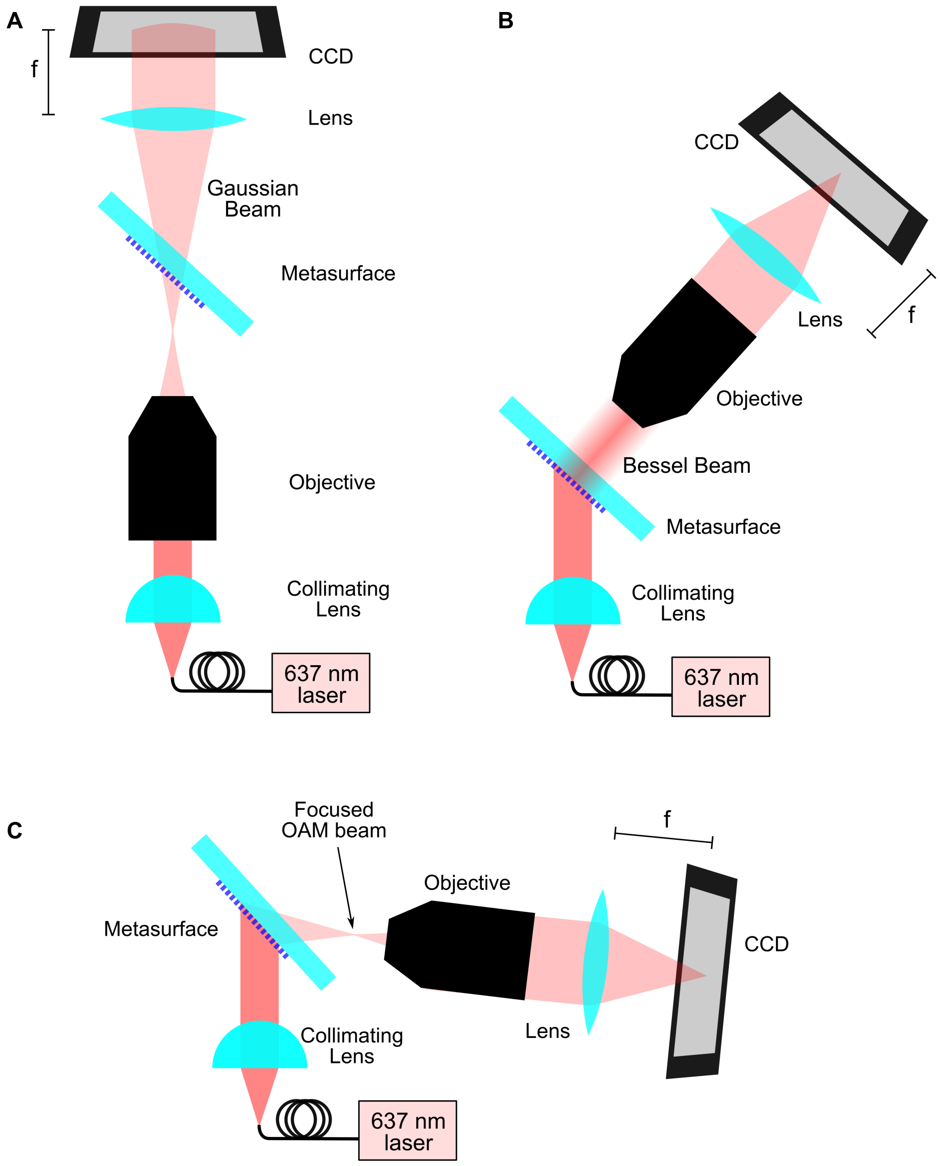

Supplementary Figure 10: **The measurement setup of device 0** (**A**) As the waist of our measurement beam exceeded the size of the metasurface, we narrowed the collimated beam by using a collimation lens of f=25.4mm and a 10x objective and projected the zeroth order onto the same CCD camera with a lens of focal distance f=5cm. (**B,C**) The SCMS was positioned on a three-axis linear stage and illuminated by a collimated beam of a laser with wavelength 637nm. The 1st and 2nd order were then imaged using a microscope-arm consisting of a 100x objective, a lens with a focal distance of 200mm and a CCD camera(sensor format 1/2”,resolution 1280 x 1024 Pixels).

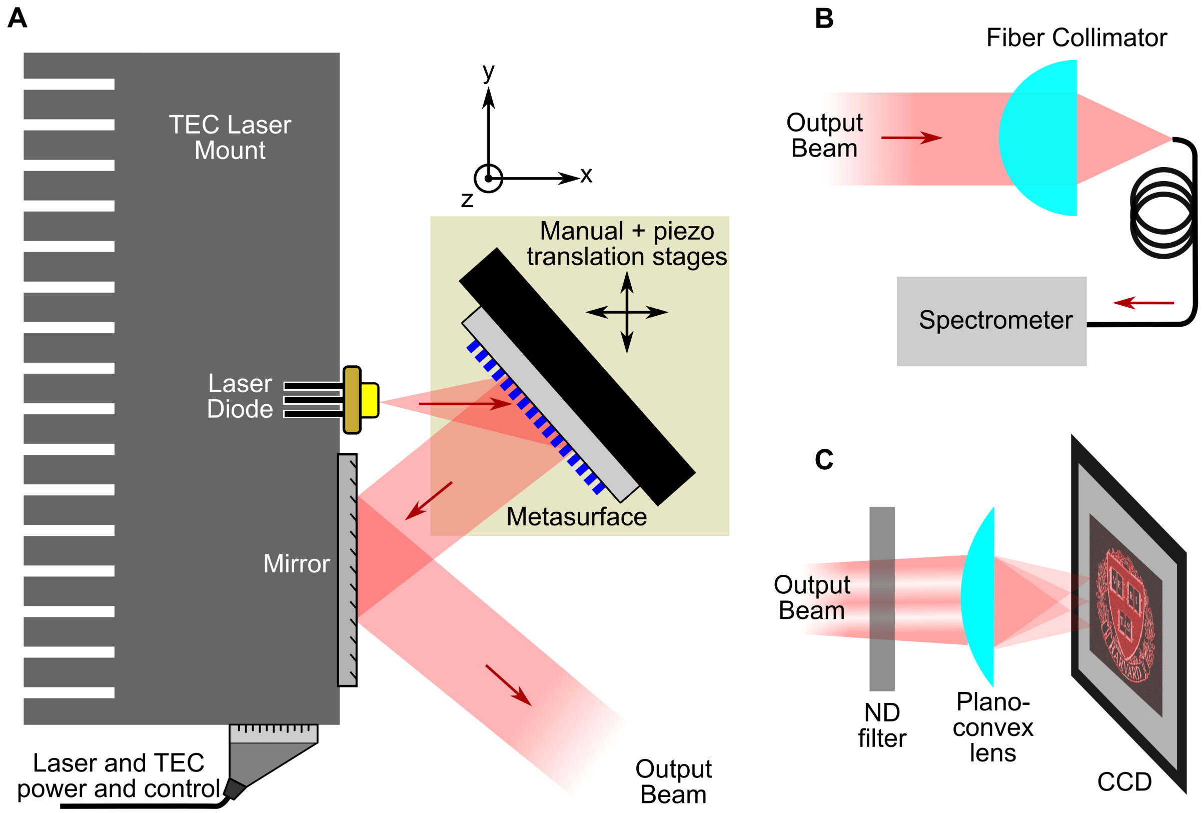

Supplementary Figure 11: **The MECL setup** (**A**) The laser assembly comprising the laser diode mounted on a TEC cooled mount, the metasurface and a mirror to reflect the output beam away from the mount. (**B**) Spectrum acquisition using a fiber-coupled grating spectrometer. (**C**) Hologram imaging setup comprising a CCD, a lens (converting far field to a focused image on the CCD) and an neutral density (ND) filter (to avoid damages to the CCD).
